# Anterior Cruciate Ligament Tear Detection Based on T-Distribution Slice Attention Framework with Penalty Weight Loss Optimisation

**DOI:** 10.3390/bioengineering11090880

**Published:** 2024-08-30

**Authors:** Weiqiang Liu, Yunfeng Wu

**Affiliations:** 1 School of Computer Science, Minnan Normal University, Zhangzhou 363000, China; lwq2688@mnnu.edu.cn; 2 Key Laboratory of Data Science and Intelligence Application, Fujian Province University, Zhangzhou 363000, China; 3 School of Informatics, Xiamen University, 422 Si Ming South Road, Xiamen 361005, China

**Keywords:** anterior cruciate ligament, slice attention, penalty weight loss, deep learning, convolutional neural network

## Abstract

Anterior cruciate ligament (ACL) plays an important role in stabilising the knee joint, prevents excessive anterior translation of the tibia, and provides rotational stability. ACL injuries commonly occur as a result of rapid deceleration, sudden change in direction, or direct impact to the knee during sports activities. Although several deep learning techniques have recently been applied in the detection of ACL tears, challenges such as effective slice filtering and the nuanced relationship between varying tear grades still remain underexplored. This study used an advanced deep learning model that integrated a T-distribution-based slice attention filtering mechanism with a penalty weight loss function to improve the performance for detection of ACL tears. A T-distribution slice attention module was effectively utilised to develop a robust slice filtering system of the deep learning model. By incorporating class relationships and substituting the conventional cross-entropy loss with a penalty weight loss function, the classification accuracy of our model is markedly increased. The combination of slice filtering and penalty weight loss shows significant improvements in diagnostic performance across six different backbone networks. In particular, the VGG-Slice-Weight model provided an area score of 0.9590 under the receiver operating characteristic curve (AUC). The deep learning framework used in this study offers an effective diagnostic tool that supports better ACL injury detection in clinical diagnosis practice.

## 1. Introduction

**Figure 1:**
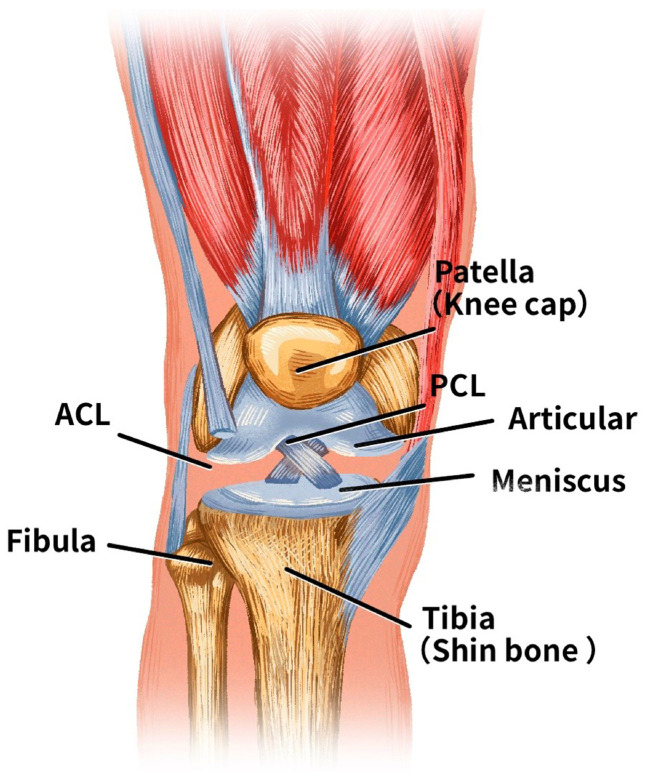
Anatomical structure of a knee joint.

The knee joint is a hinge joint that is stabilised by four main ligaments [1,2]. As shown in Figure 1, these four ligaments are critical structures that connect the bones and control joint motion. Two major ligaments are located inside the knee, and the lateral collateral ligament is located on each side of the knee. The anterior cruciate ligament [3] (ACL) and the posterior cruciate ligament [4] (PCL) cross each other inside the knee. Both ligaments anchor from the femur to the tibia, with one end attached to the femur and the other to the top of the tibia. The ACL plays a crucial role in stabilising the knee joint, and it is also the most commonly injured ligament in the human body in high-impact sports [5]. Historically, ACL injuries have been highlighted due to their long-term impact on both athletes and the general population. Unlike meniscal or collateral ligament injuries, ACL tears severely compromise knee stability and often require surgery and prolonged rehabilitation. These injuries also increase the risk of post-traumatic osteoarthritis. With increased participation in sports, especially activities involving pivoting and sudden changes in direction, ACL injuries have become more common, further highlighting their clinical importance. When ligaments are torn, surgery is usually required for treatment [5].

Anterior cruciate ligament (ACL) tears are a common type of knee injury. With the rising health awareness and increased participation in sports, the incidence of ACL injuries has been steadily increasing in recent years [6]. Research has shown that ACL injuries can lead to significant knee joint instability and significantly reduce patients’ quality of life [7,8,9,10]. Historically, ACL injuries have received particular attention due to their serious impact on long-term knee function. Unlike other sports-related injuries, such as meniscal tears or muscle strains, ACL injuries are notorious for causing chronic instability, requiring surgery and prolonged rehabilitation. ACL tears also increase the likelihood of early-onset osteoarthritis, which affects both athletes and non-athletes. These factors have made ACL injuries a major focus of orthopaedic research and treatment innovation. Therefore, prompt and effective diagnosis and treatment of ACL injuries are essential to mitigate these adverse effects.

In clinical applications, medical imaging approaches such as computed tomography (CT), magnetic resonance imaging (MRI), and ultrasound are commonly used for identifying ACL injury types [11,12,13]. Particularly, CT and ultrasound are the most challenging for accurate detection. As a result, MRI has become an important method for clinicians to investigate ACL injuries. MRI offers significant advantages in the diagnosis of knee disease because it effectively shows morphological changes in bones and joints, as well as tissue composition. It is the most commonly used and essential imaging modality for the evaluation of ACL injuries [14]. The development and application of deep learning-based models to MRI interpretation has further enhanced the potential for accurate and rapid detection of ACL injuries. In particular, deep learning models trained on MRI datasets such as MRNet have demonstrated the ability to provide automated, accurate, and reliable diagnostic support for ACL tears, significantly improving the overall diagnostic process in clinical settings.

Recently, advanced deep learning techniques in medical imaging have been extensively utilised for the detection of ACL tears [15,16,17,18,19,20,21]. Bien et al. [22] developed the knee MRNet dataset and used transfer learning from ImageNet to train nine sets of AlexNet networks on MRI images of ACL tears, meniscal tears, and other types of tears from three different views. Their results were validated using the external KneeMRI created by Štajduhar et al. [23]. Awan et al. [24] extended the ResNet14 framework using the KneeMRI dataset to identify the ACL injuries by considering a hybrid class balancing strategy for overcoming the class imbalance and the real-time data augmentation. Tsai et al. [25] proposed a lightweight model, named ELNet, which employs multi-scale normalisation and blur pooling techniques to detect ACL tears using the KneeMRI dataset. Dunnhofer et al. [26] used a pyramid feature model to effectively extract localised regions of interest (ROI) in each MRI slice. In addition, Belton et al. [27] implemented spatial attention and feature concatenation to better capture critical features within each MRI slice. These studies highlight the growing role of deep learning in ACL detection, showing promise in both reducing time to diagnosis and improving overall accuracy.

However, the key challenges of slice filtering and the exploitation of inter-class interactions arise when modelling the ACL injury diagnostic tools. Most studies either use whole data sequences that contain noise, or focus only on ROI-labelled slices with physician annotations, often ignoring the correlation and prior information between slices. In addition, these studies typically highlight individual slices and overlook the relationships between them. Furthermore, previous research often relies on classical cross-entropy loss for deep network classification, neglecting the specific inter-class relationships inherent in ACL injury tasks. To address these issues, this research proposes a novel T-distribution slice filter framework for MRNet slice selection, which effectively mitigates the slice filter problem. Current MRNet slice selection techniques often struggle to accurately identify the most relevant slices, leading to misclassification and reduced diagnostic accuracy. A key challenge is that in a series of ACL injury slices, the middle slices are typically more informative than the slices at either end. To exploit this, the T-distribution slice filter framework uses a probability-based approach to assign different weights to slices based on their position. By applying a T-distribution, higher weights are assigned to middle slices and lower weights to end slices based on the estimated mean and variance of slice positions. This method prioritises critical slices for ACL tear detection while filtering out irrelevant slices, improving overall accuracy. In addition, a penalty weight loss function is introduced to improve the inter-class connectivity of ACL tears. This loss function penalises misclassifications more heavily, thereby encouraging the model to learn more distinct and reliable features of ACL tears across different classes. As a result, this approach provides a more flexible and accurate diagnostic method for ACL injury detection. Given the increasing reliance on automated systems in medical diagnostics, the combination of slice filtering, T-distribution weighting, and penalty-weighted loss represents a significant advancement in ACL detection using deep learning models, ensuring both robustness and clinical accuracy.

This study uses deep learning techniques to effectively model MRI images using the MRNet dataset. The performance of the MRNet dataset is improved by employing a T-distribution slice filter model and incorporating a penalty weight loss function. To facilitate wider clinical application in artificial-intelligence-assisted diagnosis, this work can be extended to include ACL data from additional hospitals. The main contributions of this work are as follows:

- Development of a T-distribution slice filter: A T-distribution slice filter model was trained on the MRNet dataset to optimise slice selection for anterior cruciate ligament (ACL) imaging.
- Advanced loss function: Traditional cross-entropy loss was improved by integrating a penalty weight loss function. This modification accounts for previously unrecognised relationships between ACL categories, thereby improving the diagnostic performance of the model.
- Validation and performance: Experimental results using different backbone networks showed significant improvements with the proposed diagnostic framework. This framework provides an effective tool for ACL diagnosis to assist various clinicians in clinical settings.

These contributions directly address the challenges outlined in the introduction. The T-distribution slice filter effectively mitigates the problem of selecting relevant MRI slices by assigning higher weights to critical intermediate slices and lower weights to less informative end slices. In addition, the penalty weight loss function enhances model performance by improving inter-class discrimination of ACL tears, resulting in more accurate diagnoses. Together, these innovations result in a more accurate and efficient diagnostic tool for ACL injuries.

The rest of the paper is structured as follows: Section 2 gives a brief overview of related existing works. Section 3 describes the details of our proposed method. Section 4 provides detailed descriptions of the dataset materials and experimental procedures. Section 5 discusses the results in comparison with the state-of-the-art deep learning models. Finally, the conclusions of the study are presented in Section 6.

## 2. Previous Related Works

### 2.1. ACL Diagnosis Model

Bien et al. [22] compiled a dataset of 1370 MRI scans from the Stanford University Hospital Center, which is currently the largest publicly available database of KneeMRI. This dataset includes images from three views: sagittal, coronal, and axial. Bien et al. [22] used the AlexNet network with pre-trained parameters from ImageNet to develop nine different models, each trained to detect ACL tears, meniscal tears, and other abnormalities in the MRI images from these three views.

Belton et al. [27] built on the work of Bien et al. [22] by using ResNet18 as a backbone network to analyse three-view MRI data in three different ways. First, Belton et al. [27] fed the data into separate ResNet18 networks for feature extraction. The feature maps from the three views were then concatenated along the slice dimension and passed through a fully connected layer for classification. This method effectively integrates features from all three views. Second, instead of concatenating the feature maps directly, the second approach involves pooling and dimensionality reduction to produce three 1000-dimensional embedding vectors. These vectors are then concatenated into a single 3000-dimensional vector for classification. This method also achieves feature fusion, but by a different mechanism. Finally, the third approach is similar to the method used by Bien et al. [22], where the confidence scores of each view are computed separately. These scores are then combined using logistic regression to generate the final confidence and prediction. This approach essentially mirrors the model fusion strategy used in the study of Bien et al. [22].

Compared to these previous methods, the proposed T-distribution slice filter framework provides a more refined approach to slice selection by exploiting the prior knowledge that middle slices are more informative than those at the ends. Unlike Bien et al. [22] and Belton et al. [27], which rely on basic feature concatenation and logistic regression, the T-distribution model improves the identification of critical slices through position-based weighting, thereby enhancing the diagnostic performance for ACL tears. In addition, by integrating a penalty weight loss function, the proposed method ensures better inter-class connectivity, which is not explicitly addressed by the previous approaches.

### 2.2. Attention Mechanism

Attention mechanisms [28] have their origins in psychology, where research on cognitive behaviour has shown that people do not process all input information simultaneously. Instead, people selectively focus their attention on what is most salient. This principle inspired the development of attention mechanisms in neural networks. In practice, attention mechanisms are often used in large-scale classification tasks to improve the performance of convolutional neural networks. They do this by highlighting important features and suppressing irrelevant ones, thereby improving the overall effectiveness of the model.

The channel attention mechanism evaluates the importance of each channel in a network to generate attention in the channel domain. Hu et al. [29] implemented this mechanism in SeNet through a three-step process: squeezing, exciting, and attending. This approach involves first squeezing feature dimensions, then exciting features based on their importance, and finally attending to the original features according to their refined dimensionality.

Woo et al. [30] introduced the convolutional block attention module (CBAM), which incorporates both spatial and channel attention mechanisms. Their approach involves a simple but effective CNN attention module that combines attention across both spatial and channel dimensions to generate a comprehensive attention map.

In addition to traditional channel and spatial attention mechanisms, slice attention is also crucial in medical image classification. Slice attention focuses on the relationships between different slices of the data. Studies of Fu et al. [31], Zhang et al. [32], and Yu et al. [33] have shown that slice-based attention mechanisms are highly effective in brain MRI segmentation. In addition, Tao et al. [34] and Belton et al. [27] introduced inter-slice contextual attention and intra-slice spatial attention mechanisms to improve lesion detection. These mechanisms improve model performance while using fewer slices, thereby optimising the diagnostic process.

In comparison, the proposed method not only incorporates slice attention, but also improves slice selection by assigning different weights to slices based on their position using a T-distribution. This is a more targeted approach that specifically addresses the problem of selecting the most informative slices, which is not directly addressed by prior attention mechanisms. The T-distribution slice filter framework ensures that the middle, more informative slices are prioritised, leading to improved diagnostic accuracy in ACL tear detection. This focused approach offers an advantage over general attention mechanisms that treat all slices equally without exploiting prior knowledge of slice importance.

## 3. Deep Learning Model with Slice Attention Module and Penalty Weight Loss Function

### 3.1. Deep Learning Framework

**Figure 2:**
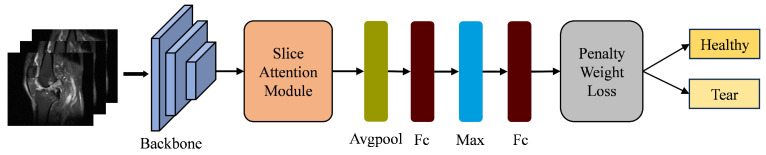
The framework of the deep learning model incorporating the slice attention module and penalty weight loss function.

As shown in Figure 2, the proposed framework consists of three main components: a T-distribution slice attention module, a backbone network, and a penalty weight loss function. The T-distribution slice attention module is crucial for analysing ACL slices that carry significant prior knowledge. These slices have high frequencies in the middle and lower frequencies at the ends of the data distribution. Therefore, the T-distribution model is used to adaptively adjust the feature weights of the ACL slices, which serve as the core mechanism of the slice attention module. The backbone network processes the features from the slice attention module, transforming data from dimensions $N_{i}\times 3\times 320\times 320$ to a feature map with dimensions $N_{i}\times C\times H\times W$, where $N_{i}$ is the number of images for the *i*-th patient and *C*, *H*, and *W* are the dimensions of the feature map. These weighted feature maps are then fed into pooling and fully connected layers to generate feature vectors. To consolidate information from the filtered slices of the same individual, the top feature at each position is selected as the representative feature. This is achieved by applying a maximum value approach as proposed by Su et al. [35]. Finally, the penalty weight loss function is used to refine the classification results from the fully connected layers.

### 3.2. T-Distribution Slice Attention Module

**Figure 3:**
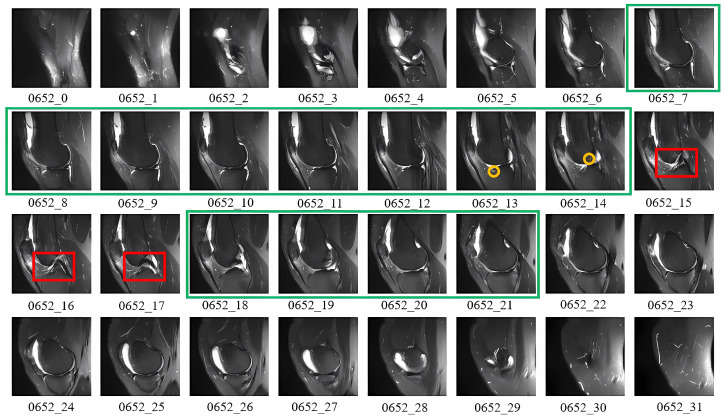
An example of direct (marked in red), indirect (marked in yellow), and potential indirect (marked in green) signs of knee joint injuries of Patient No. 0652 in the MRNet dataset.

Effective training of artificial intelligence models for ACL diagnosis relies on accurate slice filtration. ACL damage manifests itself in different parts of the knee joint and can be categorised into direct and indirect symptoms. Indirect indicators include non-specific features such as partial tears or swelling, while direct indicators, highlighted by red boxes, show structural changes. In Figure 3, layers 15–17 clearly show ACL discontinuity and significant signal dispersion, whereas layers 13–14 show indirect signs such as meniscal damage and bone marrow oedema. Diagnosis of ACL tears based on direct indicators alone may be inadequate; research by Patricia [36] and Glenn [37] highlight the importance of considering indirect signs. It is also important to note that slices at the ends of the sequence often contain irrelevant noise, whereas slices associated with ACL injuries are typically concentrated in the central region. Therefore, an effective technique must filter out noisy slices from both ends and automatically highlight those with direct and indirect signs. Given the high variability in slice content, a more sophisticated approach than traditional image classification methods is necessary to handle these challenges. Deep learning models equipped with slice attention mechanisms can more efficiently prioritise relevant slices, but they must be informed by the prior knowledge that ACL injury signs tend to concentrate in specific regions of the slice sequence. This need is particularly pressing given the paucity of annotated slices, often due to the high cost of the annotation process.

For traditional image classification tasks, common attention mechanisms include channel attention [38] and spatial attention [39]. These methods typically allow the network to learn weights automatically, without relying on prior knowledge. However, as shown in Figure 3, there is significant prior knowledge indicating that the middle slices are more critical than those at the ends. To take advantage of this prior knowledge, we can assign different weights to the slices based on their positions. This study used a T-distribution to achieve this goal. By estimating the mean and variance of slice positions using a symmetric similarity matrix, we generate a T-distribution curve for position-based weighting. This approach can ensure that middle slices receive higher weights while end slices receive lower weights, effectively incorporating the observed importance of slice positions into the model. The T-distribution slice attention mechanism provides a way to balance attention based on slice position, which is particularly suited to the structured nature of MRI data, where certain slices inherently carry more diagnostic value. Mathematically, this is expressed as follows:

(1) $$W_{i}=\frac{T(x_{i};\mu ,\nu )}{\sum_{j=1}^{S}T\left(x_{j};\mu ,\nu \right)}$$

where $T(x_{i};\mu ,\nu )$ is the probability density function of the T-distribution with mean μ and ν degrees of freedom, evaluated at slice position $x_{i}$. This ensures that slices around the central region receive higher weights than those at the ends.

**Figure 4:**
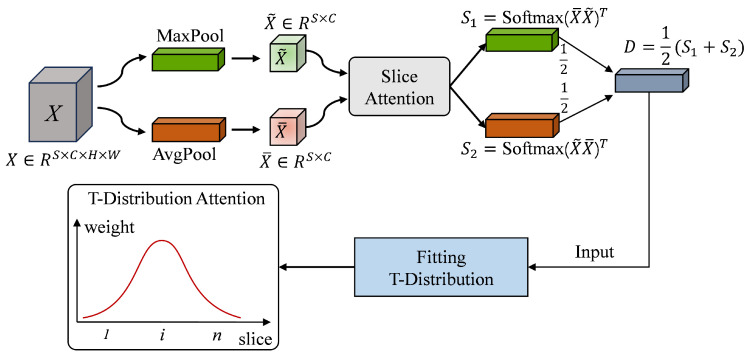
A framework of T-distribution slice attention module.

Figure 4 illustrates the framework of the T-distribution slice attention module. The high-level features *X* extracted from the original slices using the backbone network serve as input. Through global average pooling and global maximum pooling, *X* is mapped to the reshaped tensor $\bar{X}$ and $\tilde{X}$, respectively. The $\bar{X}$ and $\tilde{X}$ are then sent to the similarity measurement module, and two sets of similarity matrices $S_{1}$ and $S_{2}$ are obtained through the softmax operations. After averaging $S_{1}$ and $S_{2}$, the initial weight distribution *D* is obtained by summing the rows. It can be observed that *D* is a tensor composed of *S* scalars, where each scalar corresponds to the initial weight of a different position slice. Finally, *D* is taken as the initial weight and the importance weight of the slices is generated by the T-distribution.

In order to model the slice weights according to a T-distribution, we first estimated the mean and degrees of freedom for the positions of the slices based on their smoothed weights. For clarity, we can visualise these smoothed weights using a histogram. In this histogram, the *x*-axis represents the positions of the *S* slices (ranging from 1 to *S*), while the *y*-axis indicates the importance of these slices (ranging from 0 to 1).To generate a T-distribution, we need to compute the statistical parameters for the *x*-axis positions of the *S* slices. Specifically, we need to calculate the mean and the degrees of freedom of these positions. These statistical measures will allow us to define the T-distribution, which will then be used to assign the final importance weights to the slices. The use of the T-distribution allows the model to naturally handle the concentration of critical information around the central slices, which is consistent with observed MRI characteristics in ACL injury detection. Compared to Gaussian or uniform distributions, the heavier tails of the T-distribution provide a better fit for handling variability in slice significance, making it particularly suitable for the ACL diagnostic task. Mathematically, this can be expressed as

(2) $$T\left(x_{i};\mu ,\nu \right)=\frac{\Gamma \left(\frac{\nu +1}{2}\right)}{\Gamma \left(\frac{\nu }{2}\right)\sqrt{\nu \pi }{\left[1+\frac{{\left(x_{i}-\mu \right)}^{2}}{\nu }\right]}^{\frac{\nu +1}{2}}},$$

where Γ(·) is the gamma function. This formulation allows the model to focus on central slices while accounting for variability across the dataset.

In our normal slice attention mechanism, the T-distribution is used to iteratively adjust the weights of the slices until the loss meets the desired precision. The parameters of the T-distribution, specifically the mean and the degrees of freedom, are determined based on the statistical results obtained from this iterative process. It is important to note that the calculation of the mean and the degrees of freedom is influenced not only by the model parameters but also by the order in which the slices are input. This means that the weight distribution produced may be different for each individual. Consequently, the proposed T-distribution slice attention algorithm can dynamically adjust the importance of slices for different individuals, allowing for a more tailored and effective allocation of attention. This flexibility is crucial in medical imaging tasks, where patient variability in slice sequences can significantly affect the model’s performance. The T-distribution allows the attention mechanism to adaptively highlight relevant slices for each individual case, making it a powerful tool for improving diagnostic accuracy. As mentioned in recent research, such as that of Guzzo et al. [40] and Atmakuru et al. [41], adaptive weighting strategies are essential for dealing with heterogeneous medical datasets and varying slice importance across patients. Algorithm 1 shows the pseudocode of the T-distribution slice attention.
**Algorithm 1** T-distribution Slice Attention**Require:** 
$X\in R^{S\times C\times H\times W}$, epoch, ϵ**Ensure:** 
$M\in R^{S\times C^{\prime }\times H^{\prime }\times W^{\prime }}$ 1:Initialise $f_{1}$ and $f_{2}$, which are the feature extraction part and classification part of the network 2:**for** (i=0;i<epoch;i++) **do** 3:    $M=f_{1}\left(X\right),M\in R^{S\times C^{\prime }\times H^{\prime }\times W^{\prime }}$ 4:    $\bar{X}=\frac{1}{H^{\prime }W^{\prime }}\sum_{j=1}^{H^{\prime }}\sum_{k=1}^{W^{\prime }}M_{jk}$ 5:    X˜=max1≤j≤H′1≤k≤W′Mjk 6:    $S_{1}=Softmax\left(\bar{X}{\tilde{X}}^{T}\right),$ 7:    $S_{2}=Softmax\left(\tilde{X}{\bar{X}}^{T}\right)$ 8:    $D=\frac{S_{1}+S_{2}}{2}$ 9:    $T_{s}=\frac{1}{S}\sum_{s=1}^{S}D_{ks}$10:    $\omega_{s}=\frac{T_{s}}{\sum_{s=1}^{S}T_{s}},s=1,2,\cdots ,S$11:    $df\;=\sum_{s=1}^{S}\omega_{s}\cdot s$12:    M=W⊗M,W∼T(df)13:    $loss=f_{2}\left(M\right)$14:    **if** (loss <ϵ) **then**15:        break16:    **end if**17:    Update $f_{1}$ and $f_{2}$18:**end for**

Several steps in Algorithm 1 are critical for effective model convergence and performance by refining attention mechanisms and extracting meaningful features. In lines 4 and 5, average pooling ($\bar{X}$) captures global context, while maximum pooling ($\tilde{X}$) focuses on local salient features, balancing broad- and fine-grained information, as noted by Woo et al. [30]. Lines 6 and 7 generate similarity matrices ($S_{1}$ and $S_{2}$) via softmax, which capture correlations between slices to weight them based on importance. Line 8 computes the weight distribution *D* by summing the rows of $S_{1}$ and $S_{2}$, directing attention to the most informative slices. In lines 9 to 11, the T-distribution mechanism dynamically refines these weights, stabilising attention in the presence of noisy or redundant data. Line 12 adjusts the feature map *M* using the learned attention weights *W*, prioritising key slices during classification with iterative feedback. Finally, lines 13 to 17 implement an adaptive stopping criterion based on loss feedback, ensuring efficient convergence by preventing overfitting when the loss falls below a threshold (ϵ).

In summary, these steps integrate global and local features, refine weight distributions, and use adaptive mechanisms to ensure robust attention and prevent overfitting, which is crucial for medical image analysis tasks.

### 3.3. Penalty Weight Loss

Chen et al. [42] proposed a correlation-based penalty system to identify the varying degrees of severity of knee osteoarthritis. They emphasised that the greater the discrepancy between the predicted and observed grades, the greater the penalty should be. For example, in a five-grade knee osteoarthritis classification system, misclassifying grade 4 as grade 1 carries a different penalty than misclassifying it as grade 0. Therefore, Chen et al. [42] suggested using a penalty-weighted loss function to improve the accuracy of osteoarthritis detection.

The diagnosis of ACL tears is a classic binary classification problem, distinguishing between healthy and injured ligaments. Given the imbalance in the number of samples for different tear types, a penalty-weighted loss function may be particularly useful. To address this, a penalty weight matrix *W* is designed to capture the penalty for discrepancies between predicted and actual grades. The matrix *W* is an n×n matrix, where $W_{ij}\in W$ denotes the penalty weight for predicting grade *i* when the true grade is *j*, where $i,j\in \left\{1,2,\cdots ,n\right\}$.

For the classification of ACL tears, n=2. Each row of *W* represents the penalty vector for a given true grade *j*. Typically, the diagonal elements (where predicted and true grades match) have a penalty weight of 1. For mismatches, the penalty weight increases as the deviation from the correct grade increases. The penalty weight matrix *W*, combined with the output probabilities from the softmax layer, allows us to define a penalty-weighted loss function that adjusts the loss based on the severity of the misclassification.

(3) $$loss=\sum_{i=1}^{n}W_{ij}q_{i},$$

where *j* is the true grade of the input image and $q_{i}$ has the following definition:

(4) $$q_{i}=\left\{\begin{array}{c} p_{i},\;\;\;\;\;\mathrm{if}\;\;i\neq j\\ 1-p_{i},\;\;\mathrm{if}\;\;i=j \end{array}\right.$$

where $q_{i}$ for i=j is defined as $1-p_{i}$, because if the predicted label is of the same quality as the true label, we want to obtain the maximised output probability $p_{j}$, if the target loss is to take the minimum one; so, $1-p_{i}$ is used to make the probability optimisation objective consistent for the two positions.

To simplify Equations (3) and (4), and to allow the model to follow the output of the softmax layer, the correction to *W* is reapplied, denoted $W^{\prime }$, which is defined as follows:

(5) $$W_{ij}^{\prime }=\left\{\begin{array}{c} 0,\;\;\;\;if\;\;i=j\\ W_{ij}+1,\;\;\;otherwise \end{array}\right.$$

At this point, Equation (7) is equivalent to the following form:

(6) $$loss=\sum_{i=1}^{n}W_{ij}^{\prime }p_{i}$$

**Figure 5:**
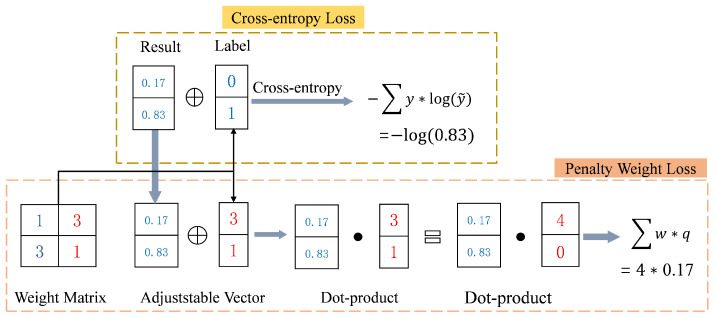
Demonstration of calculations with the penalty weight loss function, in comparison with the cross-entropy loss function.

The penalty-weighted loss function is particularly effective in addressing class imbalance, especially for categories with fewer samples, such as the tear category. To effectively address this issue, our study applied the penalty-weighted loss function to train the ACL diagnostic model. The core process of this approach is illustrated in Figure 5.

## 4. Experiments and Results

This section outlines the materials and methods used in this study. Section 4.1 provides a detailed description of the MRI image dataset and the preprocessing steps applied to the data. Section 4.2 describes the experimental setup and the evaluation metrics used. Finally, Section 4.3 presents the experimental results and provides a detailed analysis.

### 4.1. MRNet Dataset and Preprocessing

The dataset used in this study is the MRNet dataset from Stanford University Medical Center [22]. It is the largest publicly available database of knee MRI scans and includes 1370 knee MRI scans (mean age 38.0 years; 801 [58.5%] male and 569 [41.5%] female patients) performed between 1 January 2001 and 31 December 2012. The MRNet dataset includes 1008 (80.6%) abnormal examinations, of which 262 (20.9%) are related to ACL injuries. Each examination consists of a sequence of MRI slices in three orientations, with each orientation containing between 17 and 61 slices. Each slice is a grey-scale image with a resolution of 256×256 pixels.

The dataset was corrected for inhomogeneous pixel intensities in the MRI sequences using a histogram equalisation technique to standardise pixel intensity levels [43] prior to publication. Stratified random sampling was used to create the test set of 120 cases. This sampling method ensured that the test set contained at least 50 positive cases of ACL injury, thus providing a balanced representation of the data.

### 4.2. Experimental Design and Evaluation Metrics

#### 4.2.1. Experimental Design

To validate the effectiveness of our proposed method, we compare it to several baseline models, including ResNet [44], DenseNet [45], VGG [46], GoogLeNet [47], MobileNet [48], and EfficientNet [49]. These models serve as benchmarks for slice filtering, with specific modifications applied to their loss functions. First, we introduce the T-distribution slice filter framework, which automatically generates a slice filter model to effectively filter the slices. This framework improves the model’s ability to select relevant slices. Second, we replace the traditional cross-entropy loss with a penalty-weighted loss function. This novel loss function allows the model to account for the relationships between classes, thereby improving classification performance. First, we introduce the T-distribution slice filter framework, which automatically generates a slice filter model to effectively filter the slices. This framework improves the model’s ability to select relevant slices. Second, we replace the traditional cross-entropy loss with a penalty-weighted loss function. This novel loss function allows the model to take into account the relationships between classes, thereby improving classification performance. Recent studies of Motwani et al. [50] and Papanastasiou et al. [51] have shown that incorporating sophisticated loss functions and attention mechanisms can significantly improve model performance in medical imaging tasks. By implementing these modifications, we aim to demonstrate the superiority of our approach in filtering slices and accurately classifying data compared to traditional baseline models.

In the MRNet dataset, Bien et al. [22] originally divided the data into training, validation, and test sets. However, the test set has not been made publicly available. In this study, we refer to the original validation set as our test set. We then split the original training set into new training and validation sets. The number of individuals in the new validation set matches that of the new test set, and the ratio of healthy to injured individuals is maintained consistently across the new splits. This results in training, validation, and test sets with ratios of approximately 8:1:1.

The setting of hyperparameters is crucial for the training of CNN models and has a significant impact on their performance. In this study, we set the initial learning rate to $1\times 10^{-5}$ and use Adaptive Moment Estimation (Adam) for optimisation. The chosen learning rate is relatively low, which helps prevent the model from converging too quickly to a suboptimal solution and allows for more stable training, especially when dealing with complex medical image data. Recent research of Iqbal et al. [52] emphasises the importance of a carefully tuned learning rate to achieve optimal performance in medical image classification. The batch size is set to 1 and the number of training epochs is 50. A batch size of 1 was chosen to efficiently process high-resolution MRI data and to ensure that memory constraints were respected. Training for 50 epochs strikes a balance between giving the model enough time to learn and avoiding overfitting. This is consistent with recommendations from recent studies, such as those of Azizi et al. [53], which suggest that careful selection of batch size and number of epochs is critical for effective model training in medical imaging applications. To ensure reproducibility, all experiments are run with a fixed random seed.

#### 4.2.2. Performance Evaluation Metrics

For a classical binary classification problem, the predictions of the statistical confusion matrix model can be used, as shown in Table 1:

In this matrix, we define True Positive (*TP*) as the number of samples predicted to be in the positive category that are actually labelled as positive. Similarly, False Positive (*FP*) is the number of samples predicted to be in the positive category that are actually in the negative category. True Negative (*TN*) is the number of samples predicted to be in the negative category that are actually in the negative category. False Negative (*FN*) is the number of samples predicted to be in the negative category that are actually in the positive category. In addition, false positive rate (*FPR*) and true positive rate (*TPR*) are defined separately as follows:

(7) $$FPR=\frac{FP}{TN+FP},$$

(8) $$TPR=\frac{TP}{TP+FN}.$$

Therefore, for a given confidence threshold in a binary classification model, the corresponding *FPR* and *TPR* can be calculated. By plotting the *FPR* on the horizontal axis and the *TPR* on the vertical axis for each threshold, we obtain the receiver operating characteristic (ROC) curve. The ROC curve is advantageous because it is independent of the distribution of positive and negative samples, providing a more objective measure of the model’s performance. When comparing the ROC curves of different models, the area under the ROC curve (AUC) is commonly used as a measure of performance. A higher AUC value indicates better model performance. Recent works of Zeng et al. [54] and Zhang et al. [55] have demonstrated the ROC curve and AUC as critical metrics for evaluating the robustness and generalisability of classification models in medical imaging, confirming their importance in our evaluation strategy.

In addition to AUC, we utilised several other binary classification metrics to evaluate the model performance. The definitions of these classification metrics are written as follows:

(9) $$ACC=\frac{TP+TN}{TP+FP+TN+FN},$$

(10) $$Precision=\frac{TP}{TP+FP},$$

(11) $$Recall=\frac{TP}{TP+FN},$$

(12) $$Specificity=\frac{TN}{TN+FP},$$

(13) $$F_{1}=2\times \frac{Precision\times Recall}{Precision+Recall}.$$

Accuracy (ACC) is the proportion of correctly predicted samples out of the total number of samples. Precision is the proportion of true positive predictions out of all samples predicted to be positive. Recall, also known as sensitivity or TPR, is the proportion of true positives correctly identified by the model. There is often a trade-off between precision and recall; improving one may reduce the other. The $F_{1}$ score provides a balanced measure by calculating the harmonic mean of Precision and Recall, representing a compromise between the two. A higher $F_{1}$ score indicates better model performance, reflecting a good balance between Precision and Recall. Recent studies of Mosquera et al. [56] and Chamlal et al. [57] highlighted the importance of these metrics in assessing the overall effectiveness of binary classifiers, especially in the context of unbalanced medical datasets.

Typically, a binary classification model uses a default confidence threshold of 0.5, where predictions greater than 0.5 are classified as positive and those less than 0.5 are classified as negative. However, the optimal threshold for some binary classification problems may differ from 0.5. To address this, the experiments in this paper use an adaptive thresholding approach. We train the model and select the epoch with the optimal AUC on the validation set as the final model. For this epoch, we calculate the confidence threshold that achieves the highest accuracy value on the validation set. This threshold is then applied to the test set, allowing us to construct the confusion matrix and calculate the final five performance metrics. This adaptive approach is consistent with recent practices in model evaluation, as discussed in the works of Abbasian et al. [58] and Akkem et al. [59], and ensures that the model is evaluated under conditions that best reflect its real-world performance.

### 4.3. Experimental Result Analysis

To evaluate the effectiveness of T-distribution slice screening and penalty-weighted loss, this section performs ablation experiments using the MRNet dataset. In these experiments, torn ACL samples are consistently classified as positive instances, while healthy samples are classified as negative instances. To ensure a fair comparison, all experiments use the same data partitioning scheme and identical basic hyperparameter settings.

The ablation experiments on the MRNet dataset focused on evaluating the slice filter and penalty-weighted loss techniques, using the ResNet18 network as a reference model. The results of these experiments are summarised in Table 2. In the table, “Weight” represents the penalty-weighted loss and “CE” represents the cross-entropy loss. “Slice” indicates the use of slice attention filtering. The ResNet18-Slice-CE model, which incorporates slice filtering, consistently outperforms the ResNet18-CE model on all six key metrics, including a notable 0.034 improvement in the $F_{1}$ score. Similarly, the ResNet18-Weight model, which uses penalty-weighted loss, outperforms the ResNet18-CE model on all six metrics, with an impressive 0.042 gain on the ACC metric. When both techniques are combined, as in the ResNet18-Slice-Weight model, the performance improvements are even more pronounced. This model achieves a remarkable 0.05 improvement in AUC over the baseline ResNet18-CE model, along with significant improvements in other metrics. These results demonstrate the effectiveness of the proposed framework in improving model performance.

**Figure 6:**
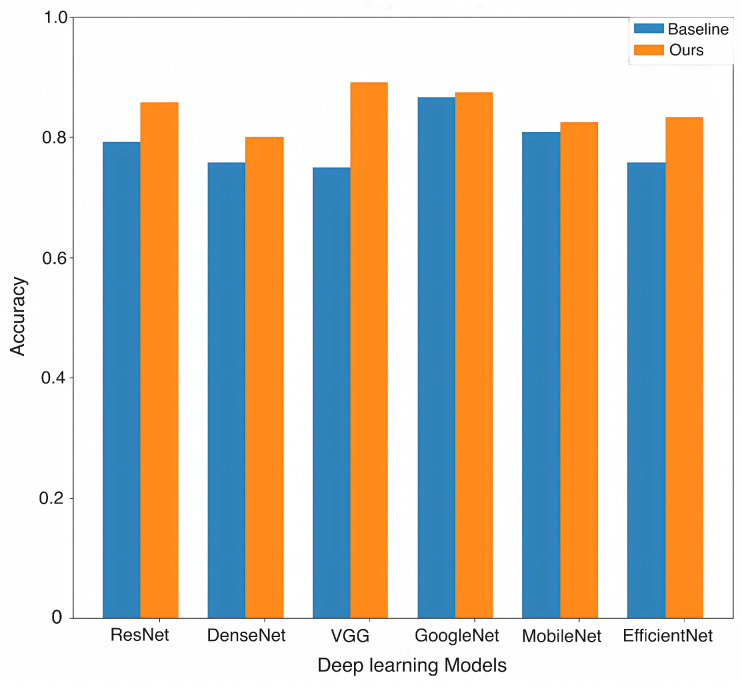
Growth in accuracy results of the deep learning backbone networks: ResNet, DenseNet, VGG, GoogleNet, MobileNet, and EfficientNet.

We evaluated the slice filter and penalty-weighted loss across different backbone networks. The results, as shown in Table 3, indicate that the incorporation of these modules generally improved performance across different backbone networks. As shown in Figure 6, the accuracy gains were significant: 0.0666 for ResNet, 0.0417 for DenseNet, 0.1417 for VGG, 0.0083 for GoogLeNet, 0.0167 for MobileNet, and 0.075 for EfficientNet. Among these results, the VGG achieved the highest accuracy of 0.8917. In addition, the VGG showed significant improvements in several metrics: precision increased by 0.1382, recall by 0.1489, specificity by 0.0757, and $F_{1}$ score by 0.1486. These improvements further validate the effectiveness of our proposed framework.

In terms of computational efficiency, as shown in Table 4, our method showed minimal increases in the number of parameters and floating point operations (flops) across all backbones. Specifically, for ResNet, DenseNet, VGG, GoogLeNet, MobileNet, and EfficientNet, the parameter increase was consistently 0.03 K, while the Flops increase was only 0.01 K per epoch, reflecting the lightweight nature of our proposed modifications. This slight increase in computational cost resulted in slightly longer training times per epoch, ranging from 13.09 s for ResNet to 11.70 s for EfficientNet, but the overall performance improvements justify these small trade-offs. Notably, the VGG network, despite having the highest number of parameters and flops, still maintained a reasonable increase in training time of only 9.68 s per epoch, further supporting the efficiency of our method.

In summary, the proposed approach not only improves model accuracy and performance metrics, but also maintains computational efficiency with minimal additional overhead, making it suitable for large-scale applications.

**Figure 7:**
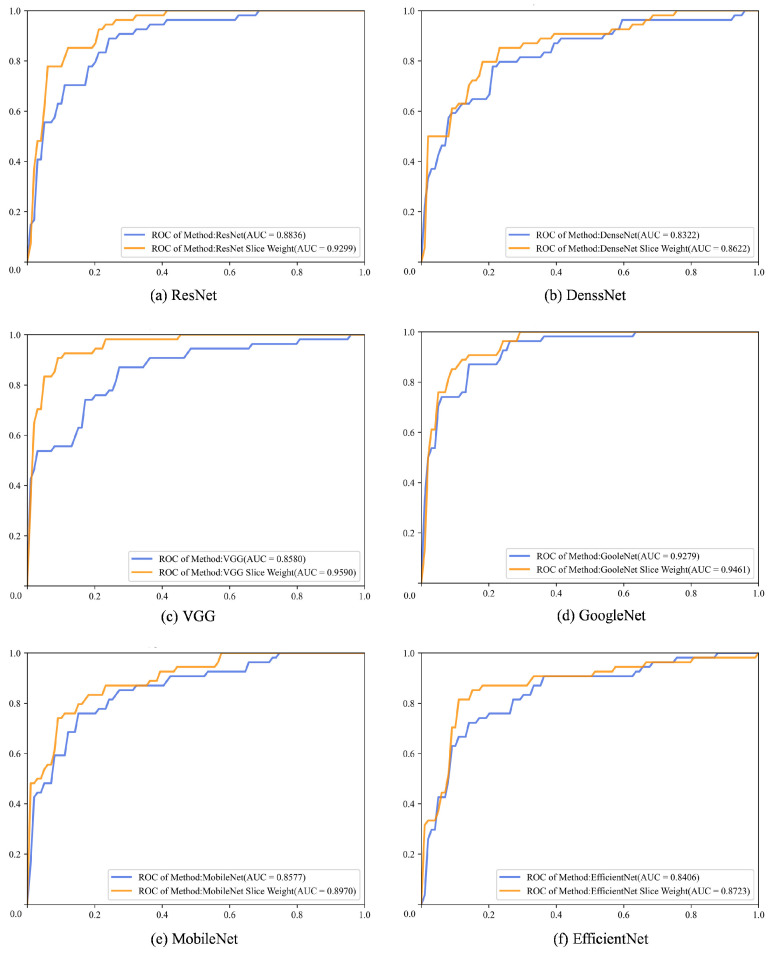
ROC graphic plots of the ResNet, DenseNet, VGG, GoogleNet, MobileNet, and EfficientNet deep learning backbone networks.

The experimental results were further analysed using ROC curves and AUC values. According to Figure 7, the ROC curves for the six popular backbone networks showed noticeable improvements, with the AUC values of our method consistently exceeding those of the baseline models. In particular, the VGG with both slice attention and penalty-weighted loss produced an AUC of 0.9590, with a significant increase of 0.101 over the standard VGG model (AUC value: 0.8580), as shown in Figure 7c.

For this work, the initial training rate should be fixed and how it is evaluated by the experiment needs to be considered. In particular, a consistent initial training rate ensures a fair comparison of the different backbone networks and models as mentioned in the experimental settings. Without a fixed initial training rate, performance variations could be attributed to the optimisation process rather than to the architectural improvements and techniques introduced, such as slice attention and penalty-weighted loss. Therefore, to ensure the reliability of the results, all experiments were performed with the same initial training rate, hyperparameter settings, and data partitioning scheme, allowing a clear evaluation of the introduced methods.

In addition, the ROC plot in Figure 7 is not consistent with previous statements. In particular, while Table 3 shows that the AUC values have improved across the different backbone networks, Figure 7 provides a visual representation of this improvement through ROC curves. The ROC curves show how well each model discriminates between classes, complementing the numerical AUC values discussed earlier. The visual representation reinforces the claim that the proposed method improves model performance in a variety of deep learning networks. Therefore, Figure 7 is essential to substantiate the results presented in Table 3, showing that our proposed framework consistently improves classification performance.

In conclusion, the method proposed in this paper has been thoroughly validated on several popular deep learning networks, showing significant performance improvements. The experimental results confirm the effectiveness of our approach for ACL tear classification. Furthermore, the successful integration of the method into different networks highlights its generalisability and consistent ability to improve the performance of different architectures.

As shown in Table 5, the MRPyrNet-MRNet-Slice-Weight model demonstrates outstanding performance, surpassing both the VGG-Slice-Weight model and the original MRPyrNet-MRNet model. With an impressive AUC of 0.9686, the MRPyrNet-MRNet-Slice-Weight model shows superior discriminative capability, excelling across key metrics such as Precision (0.8884) and Specificity (0.9848). This marked improvement over the MRPyrNet-MRNet baseline, which had an AUC of 0.9526, underscores the significant contribution of the slice attention module and penalty-weighted loss, proving its value in enhancing model performance.

The VGG-Slice-Weight model also remains highly effective, achieving a strong AUC of 0.9590 and demonstrating its robustness across all metrics. By incorporating both slice attention and penalty-weighted loss, this model delivers consistently competitive performance, showcasing its advantages in the context of medical image analysis. While the MRPyrNet-MRNet-Slice-Weight model exhibits slightly better performance in AUC and Specificity, the VGG-Slice-Weight model continues to offer a compelling balance across multiple metrics, validating the strength of its novel combination of techniques.

On the MRNet dataset, both the MRPyrNet-MRNet-Slice-Weight and VGG-Slice-Weight models demonstrate exceptional robustness and reliability. The VGG-Slice-Weight model highlights the effectiveness of combining slice attention and penalty-weighted loss for improved diagnostic accuracy. Meanwhile, the MRPyrNet-MRNet-Slice-Weight model, benefiting from the addition of the slice attention module, achieves even greater improvements, proving the efficacy of this module in enhancing overall model performance. These results set a strong benchmark for future research and practical applications in medical image analysis, illustrating the potential of these models for advanced medical diagnostic tasks.

**Figure 8:**
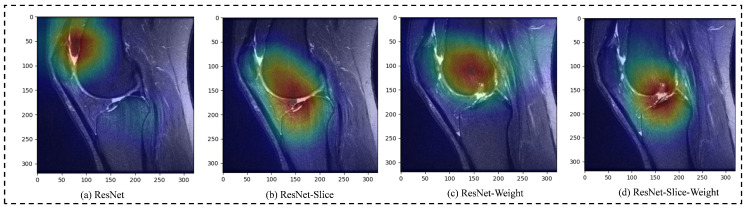
Heatmap illustrations of ResNet, ResNet-Slice, ResNet-Weight, and ResNet-Slice-Weight on the MRNet dataset.

Figure 8 shows heatmaps of MRNet images generated by the ResNet, ResNet with slice attention (denoted as ResNet-Slice), ResNet with penalty-weighted loss (denoted as ResNet-Weight), and ResNet with both of slice attention and penalty-weighted loss (denoted as ResNet-Slice-Weight). These heatmaps highlight the regions of an image that are most relevant for classification. The colour intensity reflects the degree of relevance, with lighter areas indicating greater importance.

Based on the heatmaps presented in Figure 8, the ResNet model (shown in the first column) shows minimal significant regions, while the ResNet-Slice and the ResNet-Weight model fairly to show the the location of the ACL injury. In contrast, the ResNet-Slice-Weight model, as proposed in this paper, provides clear and accurate localisation of the ACL injury. It is important to note that all models in Figure 8 are evaluated using data from the same dataset, ensuring a fair comparison between different architectures. The heatmaps clearly show that the ResNet-Slice and ResNet-Slice-Weight models significantly improve the localisation and visualisation of the ACL injury site compared to the standard ResNet model.

Furthermore, the similarity in results between the ResNet and ResNet-Weight models, as well as between the ResNet-Slice and ResNet-Slice-Weight models, can be attributed to the minimal impact of weight adjustments on the heatmaps in the absence of slice information. This suggests that the incorporation of slice-based methods is essential to improve ACL injury localisation. While weight adjustments alone have limited effectiveness in improving the standard ResNet model, their positive impact becomes evident when combined with slice information, demonstrating their value in improving model performance.

## 5. Discussion

This work represents a significant advance in the diagnosis of ACL injuries from MRNet data by combining T-distribution slice attention filtering with penalty-weighted loss. We have proposed a novel, ACL-specific model framework that exploits prior information about the importance of ACL slices. This framework effectively identifies and excludes noisy slices from the MRNet data, thereby improving the overall accuracy and reliability of the model. The integration of these filtered slices and penalty-weighted loss into the training pipelines of several backbone networks has led to significant performance improvements. In particular, the VGG slice weight model achieves an impressive AUC of up to 0.9590, highlighting its exceptional performance.

The significance of this research lies in the introduction of an unsupervised slice filtering methodology and penalty-weighted loss to assist both clinicians and computational models in the diagnosis of ACL injuries. By providing a more objective and reliable diagnostic framework, this approach reduces clinician workload and speeds up the diagnostic process, ultimately improving patient care outcomes. Furthermore, the application of these results extends beyond ACL injuries. In different medical contexts, the framework could be adapted to other musculoskeletal injuries or even organ-specific conditions where certain image slices are more clinically relevant than others. For example, this methodology could be applied to spinal MRI to detect specific disc herniations or used in cardiac imaging to focus on slices representing pathological regions. Besides the MRI data, the slice-attention mechanism could also be tailored for other imaging modalities, such as CT scans, to improve diagnostic workflows in various radiological settings. In all these cases, the penalty-weighted loss ensures that false negatives are minimised, which is critical for high-stakes medical diagnosis. In addition, the robustness of the model to different data configurations, such as different image resolutions or MRI image quality, remains a crucial area for evaluation. The performance of the proposed model on lower resolution images or in cases of suboptimal image acquisition, as often encountered in real clinical settings, should be thoroughly tested. In addition, its effectiveness on uncontrolled clinical datasets, where variability in scanning parameters and patient positioning is higher, will determine its applicability in different healthcare environments. The implementation of this approach into clinical practice is expected to significantly improve the standard of care for people with ACL injuries.

The order of content has been restructured to follow the workflow of the research more clearly. Initially, the method of ablation experiments is introduced, followed by a detailed presentation of the confusion matrix performance measures in Table 2. These measures, including accuracy, precision, recall, and $F_{1}$ score, are evaluated systematically to highlight the impact of slice filtering and penalty-weighted loss. For example, the ResNet-Slice-Weight model consistently outperforms other configurations across these metrics. The confusion matrix results not only reinforce the utility of the proposed techniques but also establish the logical progression of the research from hypothesis to validation.

Although the results are promising, there are several areas where further research is needed. Improving the generalisability of the model is crucial to ensure its effectiveness across MRI images from different hospital settings and imaging devices. In addition, the integration of slice filtering and penalty-weighted loss methods with advanced medical image processing technologies could lead to the development of a comprehensive ACL injury diagnosis system. Such integration would further improve diagnostic accuracy and support more informed clinical decision making.

In addition, ethical considerations regarding patient privacy and data security in model development and deployment require careful attention. It is essential to implement robust strategies to protect patient confidentiality and ensure compliance with data protection regulations. Such measures are critical to maintaining the integrity and trustworthiness of the diagnostic process.

In conclusion, the methodological framework presented in this paper provides a robust and effective adjunctive tool for the diagnosis of ACL injuries, with significant implications for both research and clinical practice. The results of this study could be directly implemented in AI-based diagnostic platforms in orthopaedic departments or even used to assist radiologists in various subspecialties by prioritising the most clinically relevant image slices for their review. By reducing noise and improving diagnostic speed, this framework has the potential to become an integral part of future radiology workflows, particularly in institutions dealing with high volumes of musculoskeletal imaging. By addressing the identified research directions and ethical considerations, we can pave the way for future advances in musculoskeletal imaging, ultimately transforming patient care on a global scale.

## 6. Conclusions

In summary, this thesis presents a novel method to improve ACL injury identification in the MRNet dataset by combining a T-distribution slice attention filter model with penalty-weighted loss. The methodology involves training a T-distribution slice attention module to develop an effective slice filter model. By replacing traditional cross-entropy loss with penalty-weighted loss, which exploits class relationships, the performance of the model is significantly improved. The integration of slice filtering with penalty-weighted loss results in significant performance gains, as demonstrated on six backbone networks. In particular, the VGG-Slice-Weight model achieves an impressive AUC of 0.9590. This innovative approach provides a more efficient adjunctive diagnostic strategy for ACL injury diagnosis systems. By merging slice filtering with penalty-weighted loss, the proposed framework shows great promise for clinical application and paves the way for advancements in medical imaging diagnostics.

Despite these promising results, the present study still has several limitations to be addressed in future research. First, the generalisability of the model to different clinical settings, imaging devices, and patient populations remains uncertain, and further evaluation on diverse and large datasets is required. In addition, the robustness of the model in dealing with different image resolutions and quality, especially in uncontrolled real-world clinical scenarios, needs to be thoroughly tested. Future research could explore the integration of more advanced image processing techniques, such as 3D CNNs or transformer-based models, to capture deeper spatial information across slices. Finally, future works could investigate the interpretability of the model’s decision-making process to provide greater transparency to clinicians, which could improve the confidence and facilitate wider clinical adoption.

## Figures and Tables

**Table 1:** A typical confusion matrix.

		Actual True Label	
		Positive Class	Negative Class
Predicted label	Positive class	TP	FP
	Negative class	FN	TN

**Table 2:** The experimental comparison on the MRNet dataset using ResNet.

Model	AUC	ACC	Precision	Recall	Specificity	$F_{1}$
ResNet-CE	0.8836	0.7917	0.7899	0.7921	0.7879	0.7905
ResNet-Weight	0.9186	0.8333	0.8320	0.8350	0.8182	0.8326
ResNet-Slice-CE	0.9099	0.8250	0.8243	0.8274	0.8030	0.8244
ResNet-Slice-Weight	0.9299	0.8583	0.8646	l0.8510	0.9242	0.8547

**Table 3:** Experimental results on different backbone networks (Metrics).

Backbone	Methods	AUC	ACC	Precision	Recall	Specificity	$F_{1}$
ResNet	Baseline	0.8836	0.7917	0.7899	0.7921	0.7879	0.7905
	Ours	0.9299	0.8583	0.8646	0.8510	0.9242	0.8547
DenseNet	Baseline	0.8322	0.7583	0.7592	0.7619	0.7273	0.7579
	Ours	0.8622	0.8000	0.7980	0.7980	0.8182	0.7980
VGG	Baseline	0.8580	0.7500	0.7548	0.7391	0.8485	0.7413
	Ours	0.9590	0.8917	0.8930	0.8880	0.9242	0.8899
GoogleNet	Baseline	0.9279	0.8667	0.8650	0.8670	0.8636	0.8657
	Ours	0.9461	0.8750	0.8760	0.8712	0.9091	0.8730
MobileNet	Baseline	0.8577	0.8083	0.8078	0.8039	0.8485	0.8053
	Ours	0.8970	0.8250	0.8233	0.8258	0.8182	0.8240
EfficientNet	Baseline	0.8406	0.7583	0.7643	0.7652	0.6970	0.7583
	Ours	0.8723	0.8333	0.8316	0.8316	0.8485	0.8316

**Table 4:** Average experimental results per epoch on different backbone networks (Params, Flops, and Train Time). Params and Flops columns show the average number of parameters and the average number of floating-point operations (Flops) per epoch, respectively. Params_Delta shows the absolute difference in parameters between different methods. Flops_Delta shows the absolute difference in Flops between Ours and Baseline, with Flops represented in thousands (K). Train_time shows the average training time per epoch for each method.

Backbone	Methods	Params	Params_Delta	Flops	Flops_Delta	Train_Time
ResNet	Baseline	11,690.51 K	-	113,240.46 K	-	249.56 s
	Ours	11,690.54 K	0.03 K	113,240.47 K	0.01 K	262.09 s
DenseNet	Baseline	7979.86 K	-	179,849.75 K	-	267.25 s
	Ours	7979.89 K	0.03 K	179,849.76 K	0.01 K	280.33 s
VGG	Baseline	132,869.84 K	-	470,390.17 K	-	276.38 s
	Ours	132,869.87 K	0.03 K	470,390.18 K	0.01 K	286.06 s
GoogleNet	Baseline	23,835.57 K	-	194,058.13 K	-	260.15 s
	Ours	23,835.60 K	0.03 K	194,058.14 K	0.01 K	273.20 s
MobileNet	Baseline	3505.87 K	-	20,297.49 K	-	266.96 s
	Ours	3505.90 K	0.03 K	20,297.50 K	0.01 K	269.77 s
EfficientNet	Baseline	1324.02 K	-	1716.29 K	-	256.38 s
	Ours	1324.05 K	0.03 K	1716.30 K	0.01 K	268.08 s

**Table 5:** Results of the VGG with both slice attention and penalty-weighted loss (denoted as VGG-Slice-Weight) in comparison with other state-of-the-art models on the MRNet dataset.

Model	AUC	ACC	Precision	Recall	Specificity	$F_{1}$
AlexNet [22]	0.8836	0.8333	0.8354	0.8384	0.7879	0.8331
ResNet-Space [27]	0.8763	0.8000	0.8000	0.8030	0.7727	0.7995
ELNET [25]	0.8072	0.7167	0.7168	0.7071	0.8030	0.7086
MRPyrNet-ELNET [26]	0.9172	0.8333	0.8402	0.8249	0.9091	0.8286
MRPyrNet-MRNet [26]	0.9526	0.8417	0.8523	0.8510	0.7576	0.8417
VGG-Slice-Weight	0.9590	0.8917	0.8930	0.8880	0.9242	0.8899
MRPyrNet-MRNet-Slice-Weight	0.9686	0.8443	0.8884	0.8443	0.9848	0.8508

## Data Availability

Restrictions apply to the availability of the data. Data were obtained from Stanford Machine Learning Group and are available at https://stanfordmlgroup.github.io/competitions/mrnet/ (accessed on 24 July 2024) with the permission of Bien et al. [22] at Stanford Machine Learning Group.
